# Clinical and molecular genetic characteristics of pediatric PFIC3 patients: three novel variants and prognosis for parental liver transplantation

**DOI:** 10.1186/s13023-025-03670-y

**Published:** 2025-04-08

**Authors:** Jiqiang Hu, Chenyu Yang, Bingqian Tan, Qiang Xiong, Ying Le, Jianyang Hu, Haoming Wang, Xiaoke Dai, Mingman Zhang

**Affiliations:** Department of Pediatric Hepatobiliary Surgery National Clinical Research Center for Child Health and Disorders, Ministry of Education Key Laboratory of Child Development and Disorders, Chongqing Key Laboratory of Pediatric Metabolism and Inflammatory Diseases, Children’S Hospital of Chongqing Medical University, Chongqin, China

**Keywords:** Progressive familial intrahepatic cholestasis type 3, ABCB4, MDR3, Cholestasis

## Abstract

Progressive Familial Intrahepatic Cholestasis Type 3 (PFIC3) is a rare inherited liver disease caused by a mutation in the ABCB4 gene, leading to dysfunction of multidrug resistance protein 3 (MDR3). The earlier the onset of PFIC3 in children is, the more severe the prognosis. The diagnosis of PFIC3 is typically based on clinical symptoms, laboratory tests, and imaging assessments, with final confirmation requiring genetic testing. The aim of this study was to investigate the associations between genetic mutations in PFIC3 and clinical features, molecular genetics, and liver histopathology to improve early recognition and understanding of this disease. By analysing the data of three children with PFIC3 who underwent parental liver transplantation, we were able to gain a deeper understanding of the complexity and diversity of the disease. With respect to molecular genetics, we identified five mutation sites in the ABCB4 gene, including three newly discovered mutations. Immunohistochemical analysis revealed reduced expression of the MDR3 protein in child 1 and no expression in child 2 or child 3, revealing an intrinsic link between the ABCB4 gene and the MDR3 protein. Histopathologically, all three patients presented with significant portal vein fibrosis or cholestatic liver cirrhosis. In conclusion, this study emphasizes the importance of molecular genetic and pathological evaluation of patients with PFIC3 mutations and elucidates the impact of these three mutations on the course of the disease in children, for whom early symptomatic treatment and early preparation for liver transplantation are options worth considering.

## Introduction

PFIC3 is a rare recessive liver disorder that falls within the category of Familial Intrahepatic Cholestasis (PFIC). PFIC is a spectrum of inherited disorders of intrahepatic cholestasis, arising from various genetic mutations. The global incidence of PFIC is estimated to be between 1 in 100,000 and 1 in 50,000 individuals [1], with PFIC3 exhibiting a relatively lower prevalence. Nonetheless, PFIC3 significantly impairs patients' quality of life and health.

PFIC3 is precipitated by mutations in the ABCB4 gene, which is located on human chromosome 7q21. This gene encompasses 75,504 bases and 28 exons [2]. The ABCB4 gene encodes MDR3, a membrane transporter protein predominantly expressed on the tubular aspect of hepatocytes. MDR3 is pivotal for the normal synthesis of bile, as it facilitates the transport of phosphatidylcholine (PC) from within liver cells to the bile [3]. Concurrently, the mixed micelles of phospholipids and bile salts within the bile aid in the emulsification of bile salts, increase the excretion of bile acids, and provide protection to biliary ductal epithelial cells against the toxic effects of bile acids [4].

As patients with PFIC3 mutations exhibit diverse genetic mutations, considerable variation in the age of onset and progression of their condition is noted. Certain patients with single-point mutations may respond positively to ursodeoxycholic acid (UDCA) therapy, whereas those with nonsense mutations typically suffer from more severe disease manifestations. These individuals often present with early symptoms and rapid deterioration of their condition, frequently necessitating liver transplantation [5]. Children diagnosed in early childhood frequently present symptoms such as jaundice, hepatomegaly, impaired liver function, and coagulopathy [5]. Cholestasis leads to the accumulation of bile constituents, including biliary toxins, bile acids, and cholesterol, which can impair liver cells and contribute to the development of liver fibrosis. Moreover, severe pruritus, a common complication of cholestasis in paediatric patients, significantly impairs quality of life and can lead to complications such as sleep disturbances. Consequently, it is essential for patients with PFIC3 to be considered on an individual basis, with tailored treatment strategies selected to enhance prognosis and improve overall quality of life.

The diagnosis of PFIC3 requires consideration of the patient's clinical manifestations, family history, and laboratory results. Patients typically present with advancing jaundice, pruritus, hepatomegaly, abnormal liver function, and coagulation disorders. Elevated concentrations of serum bile acids and other abnormal liver function indicators also serve as diagnostic cues for PFIC3. Genetic testing is crucial for confirming PFIC3, as it can identify the type of mutation in the ABCB4 gene and further illuminate the genetic basis of the disease.

Drug therapy constitutes one of the principal treatment approaches for PFIC3 patients, and commonly prescribed drugs include bile-regulating, anti-inflammatory, and antipruritic medications. They can increase the quality of life of patients by facilitating bile flow, ameliorating liver function, and alleviating symptoms of inflammation and itching. Nevertheless, in instances where the disease progresses at a faster pace or severe liver function impairment emerges, liver transplantation might be the sole treatment option. Although liver transplantation can be life-saving, it is accompanied by a multitude of risks, complications, a scarcity of donor livers, and the need for long-term immunosuppressive therapy.

In conclusion, PFIC3 is a rare hereditary liver ailment distinguished by intrahepatic cholestasis and progressive deterioration of liver function. The diagnosis is currently grounded in the patient's clinical symptoms, family history, and genetic testing. Treatment strategies include pharmacological interventions and liver transplantation, which may lead to significant complications, including acute rejection, lymphoproliferative disorders post-transplantation (PTLD), and other adverse effects. Hence, we present the case histories of three paediatric patients with PFIC3 who underwent parental liver transplantation. Through the application of morphological analysis, our objective is to augment the existing knowledge and comprehension of this disease, increase early detection rates, optimise therapeutic outcomes, and ultimately benefit patients.

## Participants and methods

### Participants

This investigation included three paediatric patients who underwent parental liver transplantation for PFIC3 at our institution between July 2020 and October 2022. All three participants fulfilled the diagnostic criteria for PFIC3: (1) the patients exhibited progressive jaundice, compromised liver function, bile acid stasis, deficiency of fat-soluble vitamins, and elevated serum γ-GGT levels; (2) imaging revealed hepatomegaly and splenomegaly, with exclusion of other conditions that could precipitate cholestasis; and(3) genetic testing ultimately confirmed an ABCB4 gene mutation in all three children. We collated patient history, physical examination findings, laboratory test results, and molecular genetic data.

### Molecular genetics

All three children were diagnosed through Next-Generation Sequencing (NGS), with genetic testing and analysis services conducted by the Beijing MyGenostics Medical Testing Institute. Specifically, patients 1 and 2 were subjected to the analysis item M019V2: Metabolic Liver Disease Gene Detection Panel V2, whereas patient 3 underwent WES016-1: Trios Whole Exome Gene Detection V6 (200X). In accordance with the guidelines of the American Society for Medical Genetics and Genomics (ASMGG), the pathogenicity of the identified mutations was assessed and initially classified into five categories: “pathogenic,” “possibly pathogenic,” “of uncertain significance,” “possibly benign,” and “benign” [6]. The molecular nomenclature followed the guidelines of the Human Genome Variation Society (HGVS, http://varnomen.hgvs.org/), and the human cDNA sequence of the ABCB4 gene was obtained from the Human Gene Mutation Database (HGMD, http://www.hgmd.cf.ac.uk).

### Hepatic histopathology

The experimental cohort comprised patients with PFIC3 (n = 3) who had undergone parental liver transplantation, whereas the control group consisted of liver specimens adjacent to hepatoblastomas in paediatric patients. The samples were fixed in formalin and subsequently cut into 4 μm thick sections via paraffin embedding for histopathological analysis. Routine histological examination was performed via Hematoxylin and Eosin (H&E) staining. A Sirius red staining kit (G1472, Solarbio, China) was used to assess the extent of liver fibrosis. The expression of CK19 in bile ducts within the liver tissue was evaluated through immunohistochemical staining. The liver tissue sections were subjected to antigen retrieval via incubation with Tris–EDTA solution (PH 9.0) for 30 min at 98 °C and blocked with 3% endogenous peroxidase (H2O2). These sections were then incubated overnight at 4 °C with a 1:500 dilution of anti-CK19 antibody (ab52625, dilution 1:500; Abcam, US). The second antibody was incubated with goat anti-rabbit IgG at room temperature for 1 h, followed by conventional hematoxylin staining after 90 s of DAB colour development, and the sections were sealed with neutral gum after dehydration and dewaxing. MDR3 immunohistochemical staining was used to analyse protein expression. The liver tissue sections were subjected to antigen retrieval via incubation with Tris–EDTA solution (PH 9.0) for 30 min at 98 °C and blocked with 3% endogenous peroxidase (H2O2). These sections were then incubated overnight at 4 °C with a 1:4000 dilution of anti-MDR3 antibody (ab272457,dilution1:4000;Abcam,US), the second antibody was incubated with a goat anti-rabbit IgG antibody at room temperature for 1 h, followed by conventional hematoxylin staining after 90 s of DAB colour development, and the sections were sealed with neutral gum after dehydration and dewaxing. Finally, the slides were scanned via a full slide scanner.

## Results

We aggregated the clinical data and laboratory findings from three distinct families (Table 1). The ages at onset were 131 months, 49 months, and 47 months, with intervals between onset and liver transplantation of 22 months, 11 months, and 4 months, respectively. All the children exhibited severe jaundice, and diagnostic imaging (ultrasound, CT, and MRCP) revealed hepatic and splenic enlargement, without evidence of cholelithiasis; other potential causes of cholestasis, including biliary atresia, primary biliary cholangitis (PBC), primary sclerosing cholangitis (PSC), viral infections, and metabolic disorders, were excluded.

**Table 1:** Clinical information and laboratory findings

Patient	Child1	Child2	Child3
The ages at onset (Months)	131	49	47
The ages at liver transplantion(Months)	153	60	51
*Clinical and examination features*			
Decompensated Cirrhosis	+	+	–
Hypersplenism	+	+	–
Anemia	+	+	+
Jaundice	+	+	+
Coagulation Dysfunction	+	+	+
Hepatosplenomegaly	+	+	+
Cholelithiasis	–	–	–
Esophageal and Gastric Varices with Bleeding	+	–	–
Growth Retardation	–	–	+
UDCA	+	–	–
Biopsy(Biliary Cirrhosis)	Stage IV	Stage IV	Stage III-IV
*Biochemical test results (preoperative)*			
TBA (μmol/L)	19.4	134	136.3
TBIL(μmol/L)	136	499.2	21.2
DBIL(μmol/L)	53.2	115.8	2.6
IBIL(μmol/L)	82.8	321.4	18.6
ALT(U/L)	126	156	177
AST(U/L)	128	697	349
γ-GGT(U/L)	218	241	249
PLT(*10^9/L)	50	72	158
NH₃(μmol/L)	31.7	80.5	28.3
*Biochemical test results (2 weeks postoperative)*			
TBA(μmol/L)	13.7	11.4	15.4
TBIL(μmol/L)	35.6	90.8	4.9
DBIL(μmol/L)	9.6	37.1	2.4
IBIL(μmol/L)	26	30.6	0
ALT(U/L)	100	64	36
AST(U/L)	37	52	23

Child 1 showed some improvement with UDCA treatment, yet the disease persisted. Throughout the disease course, due to haematochezia and acute upper gastrointestinal bleeding, emergency interventions and blood transfusions were repeatedly needed. Endoscopy revealed oesophageal and gastric fundus varices with bleeding. Children 2 and 3 did not respond significantly to UDCA treatment, and their disease progression was rapid. Both child 1 and child 2 developed hepatic decompensation and hypersplenism as their cirrhosis progressed. All three children experienced anaemia and coagulation issues. Notably, the height and weight of child 3 are less than those of children of the same age [7], indicating growth retardation.

In preoperative biochemical assessments, γ-GGT levels were notably elevated in three children, alongside total bile acid and total bilirubin. After liver transplantation, biochemical marker levels tended to increase, with biochemical examination data reaching normal levels at two weeks after the procedure. The pathological examination report following liver transplantation revealed hepatocellular hyperplasia, the formation of false lobules, swelling of hepatocytes, biliary pigmentation, focal necrosis and regeneration, compression or occlusion of hepatic sinusoids, significant expansion of the manifold area, frequent collagen deposition, dilation of small bile ducts, increased lymphocyte infiltration, and evidence of biliary cirrhosis.

### Molecular genetics

In the genetic testing reports for three children, we identified five variant sites within the ABCB4 gene (Table 2), encompassing four heterozygous variants and one homozygous variant. These variant loci are as follows: C.2493G > C (p.R831S), C.592G > A (p.A198T), C.475C > T (p.R159X), C.938C > T (p.A313V), and C.3136C > T (p.R1046X).

**Table 2:** Mutations in the ABCB4 gene

Patient	Age at genetic testing(months)	Chromosome location	Nucleotides/aminoacids	Heterozygous/homozygous	Revel software Prediction	ACMG evidence	ACMG disease analysis	Type of mutation	Report	Parental mutation status
Child1	132	chr7:8704 6817	c.2493G > C (p.R831S)	Het	deleterious	PM2/PP3	Uncertain	–	No	Unsampled
		chr7:8708 1055	c.592G > A (p.A198T)	Het	Benign	PM2/PP3	Uncertain	–	No	
Child2	52	chr7:8703 7496	c.3136C > T (p.R1046X)	Hom	Unknown	PVSI/PM3/PM2	Pathogenic	No Sense Mutation	Yes (pathogenic mutation)	Both parents are heterozygous mutation
Child3	47	chr7:8708 2321	c.475C > T (p.R159X)	Het	Unknown	PVS1/PM2/PM3	Pathogenic	No Sense Mutation	Yes (pathogenic mutation)	Father normal
		chr7:8707 6417	c.938C > T (p.A313V)	Het	deleterious	PM2/PP3	Uncertain	missense mutation		Father heterozygous mutation

According to the ACMG criteria. PVS 1 mutation leads to the complete loss of gene products and impaired gene function, which is very strong evidence of pathogenicity. PS 1–4 mutation is clearly caused by in vivo and in vitro functional experiments, which are strong evidence of pathogenicity; PM1-6 the mutation is not found in the normal control population of the ESP database, 1000 people database or ExAC database (or is a very low frequency locus in recessive genetic diseases),indicating that the incidence of this variant in the population is very low, which is moderate evidence of pathogenicity; PP 1–5 mutation is co-separated from the disease in the family lineage, which is auxiliary evidence of pathogenicity

Child 1 carries two heterozygous variants, which represent novel mutations in the ABCB4 gene. Specifically, the variant at c.2493G > C (p.R831S) involves the substitution of guanine (G) for cytosine (C) at nucleotide 2493, leading to an amino acid change. The protein function prediction software REVEL has assessed this mutation as deleterious. Conversely, the variant at c.592G > A (p.A198T) involves the replacement of guanine (G) with adenine (A) at nucleotide 592, resulting in an amino acid mutation that REVEL software has classified as benign. Considering the patient's clinical history, we hypothesize that her protein function might have been partially conserved, yet liver fibrosis and liver function impairment progressively escalated over time, culminating in the manifestation of PFIC3 disease.

Child 2 harbours a homozygous variant, C.3136C > T (p.R1046X), which involves the substitution of cytosine (C) with thymine (T) at nucleotide position 3136. This alteration results in a nonsense mutation of the amino acid, potentially leading to a complete loss of gene function. In prior disease reports, this particular homozygous variant has been associated with cholesterol gallstone disease [8]. In accordance with the ACMG guidelines, the pathogenicity of this variant was evaluated as pathogenic. In conjunction with the clinical information and pathological findings, we arrived at a definitive diagnosis of PFIC3.

The two mutation sites identified in child 3 are also heterozygous mutations. The variant C.475C > T (p.R159X) involves the transition of cytosine (C) to thymine (T) at nucleotide 475, leading to a nonsense mutation of amino acids and, potentially, a complete loss of gene function. This mutation site has been previously diagnosed as PFIC3 in disease reports [9], with the onset age of the affected child being 54 months, which is comparable to the age of onset for Child 3 in our study, 47 months. In line with the ACMG guidelines, the pathogenicity of this variant was determined to be pathogenic. The second mutation site, C.938C > T (p.A313V), changes cytosine (C) to thymine (T) at nucleotide 938, resulting in an amino acid substitution from alanine to valine at position 313, causing a missense mutation. The REVEL software predicts this mutation to be deleterious.

Among the five mutation sites identified across the three children, three are novel. In light of their clinical and genetic profiles, we hypothesize that C.2493G > C (p.R831S), C.3136C > T (p.R1046X), and C.475C > T (p.R159X) may be pathogenic. Given the potential impact of these mutation sites on MDR3 protein expression in hepatocytes, we performed MDR3 immunohistochemical staining on the liver tissues of these three children. This investigation aimed to further elucidate the effects of these mutation sites on protein expression. We hope that this analysis will provide deeper insight into how these genetic variants contribute to the onset and progression of PFIC3 disease.

### Liver histopathology results

**Fig. 1:**
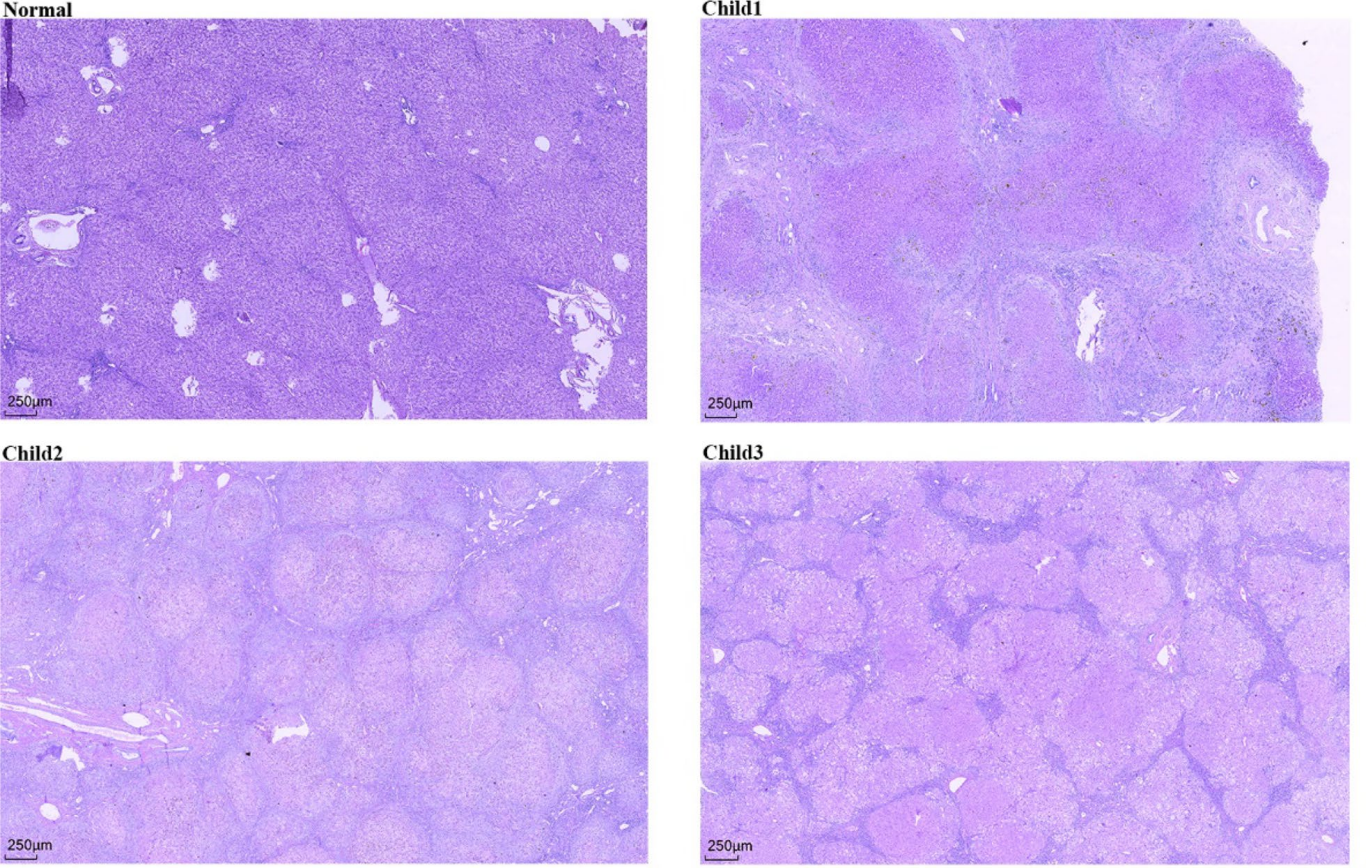
HE staining of liver tissues from children with PFlC3 (40x). HE staining of liver tissues from Child 1, Child 2, and Child 3 revealed significant fibrosis, pseudolobule formation, and bile pigment deposition. The portal areas are markedly expanded, with considerable infiltration of inflammatory cells and hepatocyte ballooning degeneration

**Fig. 2:**
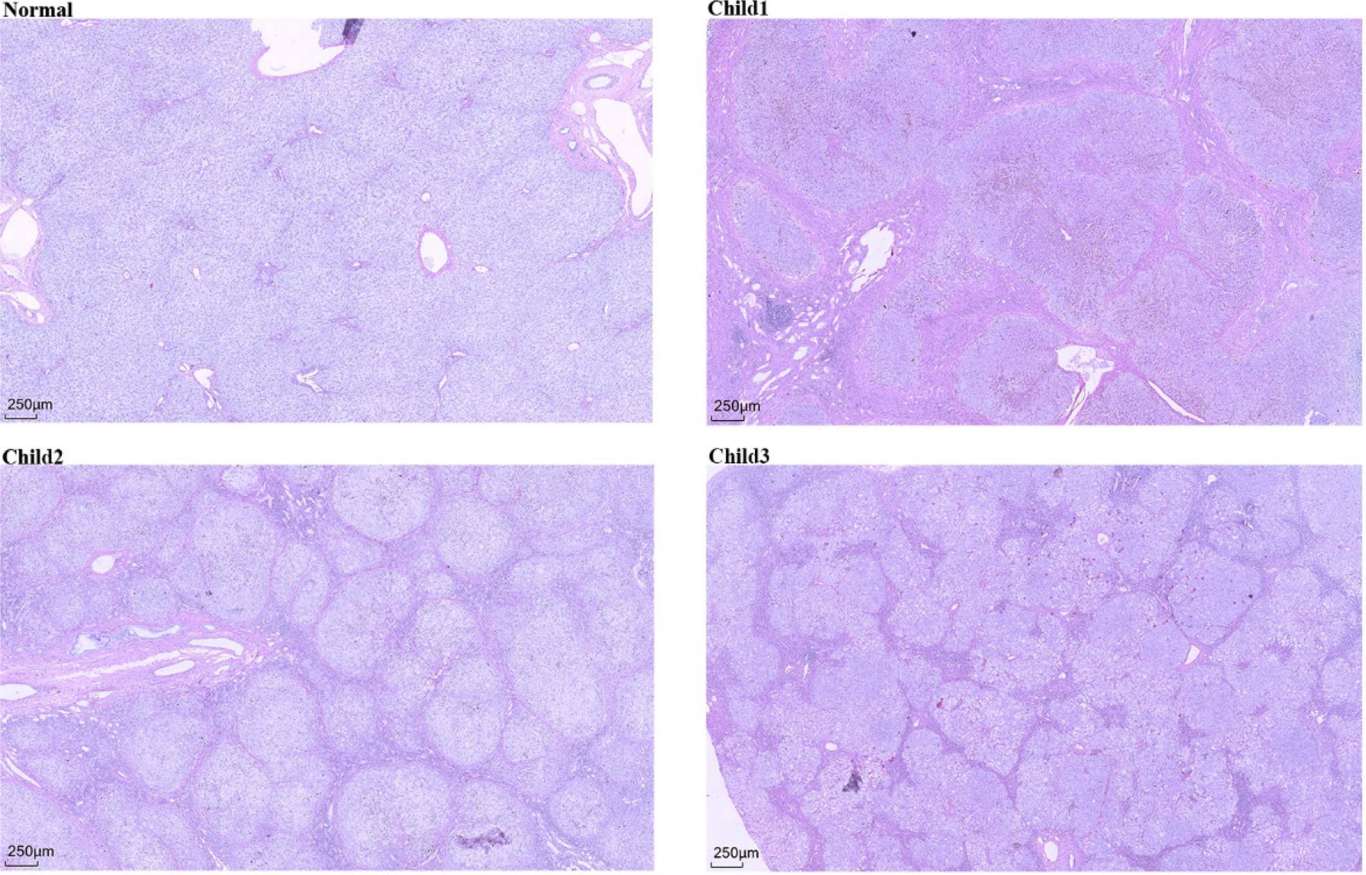
Sirius red staining (40 × magnification). Liver tissue from three children showed massive fibroplasia and pseudolobule formation

Upon histopathological examination, we observed significant portal vein fibrosis or biliary cirrhosis in all three children (Fig. 1). Inflammatory cell infiltration was predominantly concentrated in the dilated sinus areas, indicating a particularly active inflammatory response in these regions. We also noted that some hepatic sinuses were compressed or closed, which further impeded normal blood circulation to the liver. Some hepatocytes exhibited oedematous degeneration, a manifestation of liver cell damage. Additionally, in both Child 1 and Child 2, we observed biliary sludge and partial bile thrombosis within hepatocytes, suggesting severely compromised bile excretion. Sirius red staining (Fig. 2) revealed a clear presence of proliferating collagen fibres and the formation of false lobules, which are typically indicative of an active fibrosis process and significant structural alterations within the liver.

**Fig. 3:**
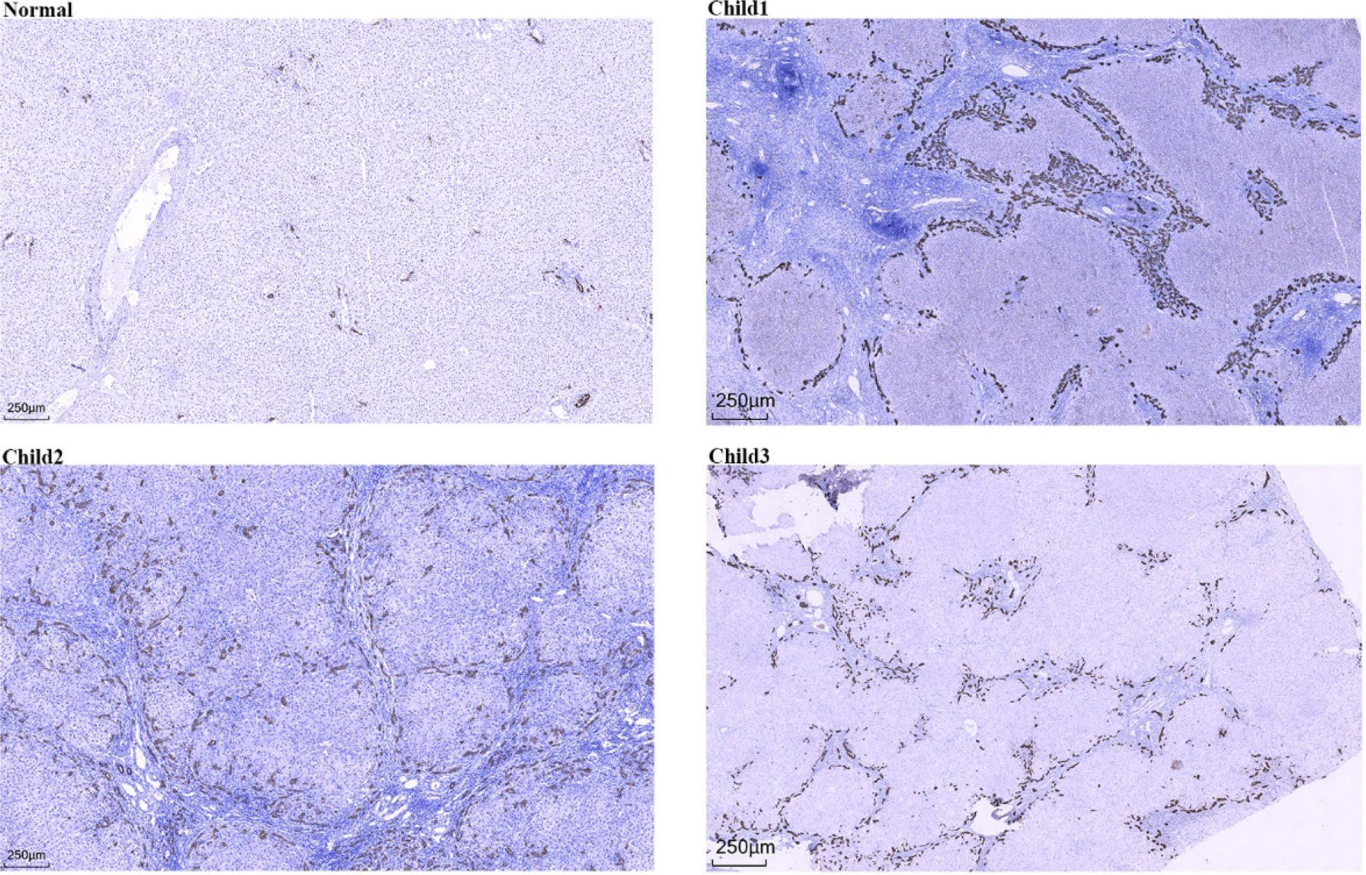
Immunohistochemical staining of CK19 in liver tissues of children with PFlC3 (40x). The image shows partial absence of bile ducts in the enlarged hilar region and active bile duct proliferation at the junction of the hilar region and the liver parenchyma. Ectopic expression of CK19 around the central vein and hepatic parenchyma was also observed in Child 2 and Child 3 patients

Bile duct injury is a prominent pathological feature of PFIC3 disease. The results of CK19 immunohistochemistry (Fig. 3), revealed that the liver tissues of the three children presented varying degrees of bile duct lesions, including inflammatory infiltration of the bile duct epithelium, loss of some bile duct epithelial cells in the sinus area, active bile duct hyperplasia within the sinus area and the edge of the liver parenchyma, and disordered bile duct arrangement. These changes suggest that, despite continuous destruction of the bile duct structure, bile duct hyperplasia is highly active at the edge of the hepatic parenchyma. This proliferation, though irregular, may lead to disordered bile duct arrangement, exacerbating the obstruction of bile excretion. In Children 2 and 3, we even observed ectopic expression of CK19 in the central vein and surrounding hepatic parenchyma, which may indicate hepatocyte transdifferentiation into bile duct-like cells, a common phenomenon in the liver during injury and regeneration.

**Fig. 4:**
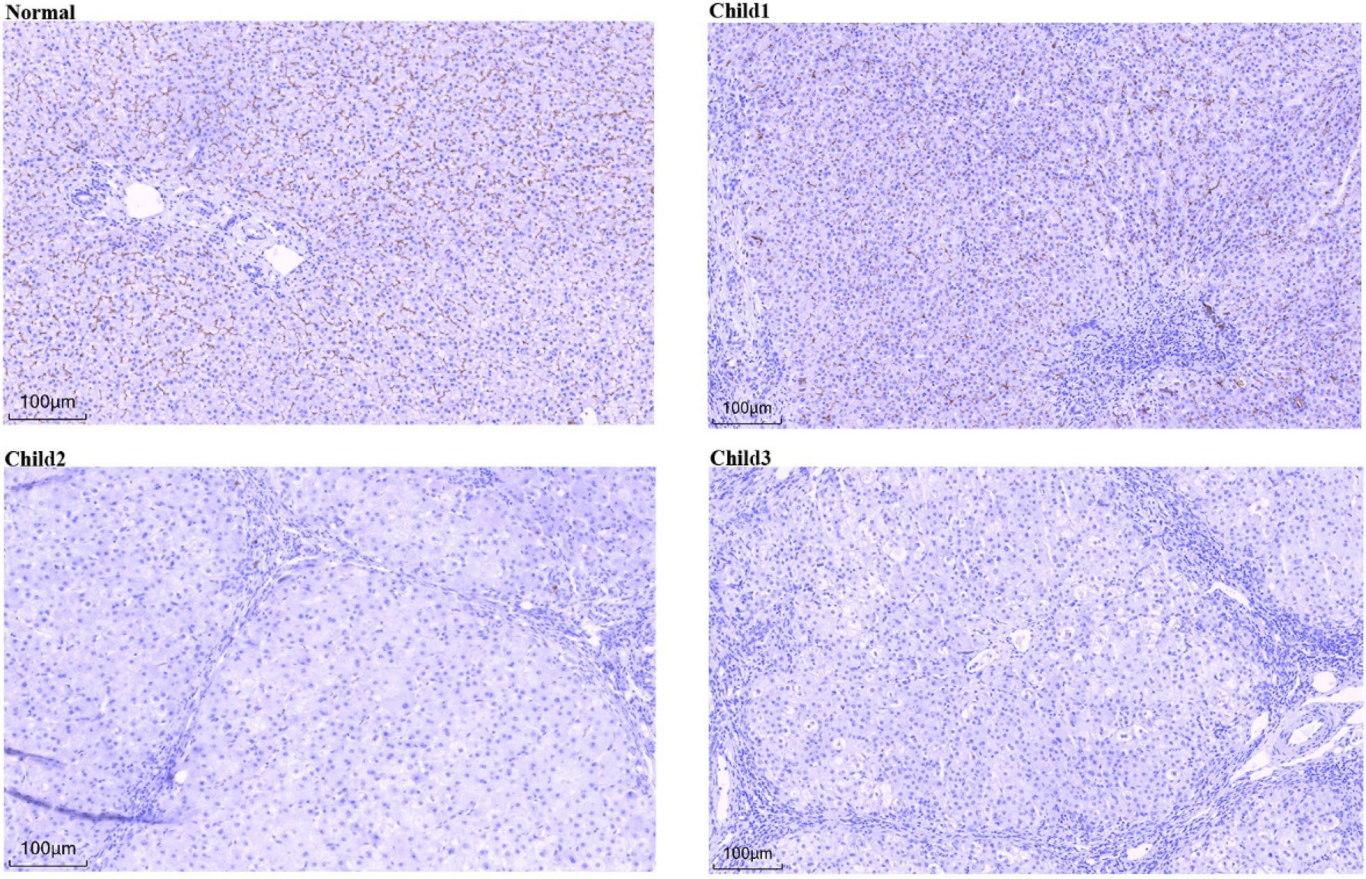
Immunohistochemical staining of the liver MDR3 protein (200x): In normal children, the MDR3 protein is expressed on the membrane side of the hepatocyte tubules. Compared wirh the normal group, Child 1's liver tissue presented reduced expression of the MDR3 protein; MDR3 protein expression was absent in the liver tissue of Child 2 and Child 3

The immunohistochemical results of MDR3 (Fig. 4), revealed that the expression of MDR3 in the liver tissue of child 1 was notably lower than that in the normal control group, indicating a decreased content of phospholipids in the bile. This reduction in phospholipids increased bile toxicity, further damaging the bile duct epithelium and liver cells. This finding corroborates the reason for the slower clinical disease progression in child 1 than in child 2 and 3. MDR3 expression was significantly absent in the liver tissue of child 2 and 3, suggesting that their bile lacks phospholipids, which may explain their faster disease progression and earlier onset.

## Discussion

Our study presents the clinical, genetic, and liver pathological characteristics of three children with PFIC3 from three distinct families, with the aim of enhancing the understanding of the disease and improving the diagnosis and treatment of patients.

PFIC3 is caused by a genetic mutation that results in a dysfunctional variant of the MDR3 protein, with the age of onset varying from childhood to adulthood. The younger the age of onset, the faster the disease progression and the more pronounced the clinical features. In our report, all three children presented typical features of PFIC3, including progressive jaundice, pruritus, and impaired liver function. The disease progressed rapidly, necessitating LDLT operations at 22 months, 11 months, and 4 months. Child 1 developed oesophageal and gastric varices complicated by severe upper gastrointestinal bleeding due to the progression of liver fibrosis, and his condition improved following emergency treatment and blood transfusion. Child 2 also required multiple transfusions for anaemia and coagulation dysfunction. Laboratory tests revealed continuously elevated levels of ALT, AST, TBA, DBIL, and γ-GGT in all the children, confirming the presence of jaundice and liver function impairment associated with PFIC3. Previous studies have demonstrated [1, 10, 11] that serum γ-GGT levels are elevated in PFIC3 patients, which contrasts with the normal serum γ-GGT levels observed in PFIC1 and PFIC2 patients. In patients with unexplained cholestasis and elevated γ-GGT levels, PFIC3 should be considered after other causes of cholestasis are excluded [10, 12].

Child 1 experienced a certain degree of remission following UDCA treatment, suggesting that the MDR3 protein in his liver tissue retained some functional capacity. However, his condition subsequently deteriorated, indicating that UDCA treatment could not fully ameliorate his condition. In contrast, Children 2 and 3 did not respond to UDCA treatment, and their disease progressed rapidly, suggesting that the MDR3 protein in their liver tissues may have completely lost its function. Liver biopsies performed on all three patients indicated biliary cirrhosis (stages IV, IV, and III-IV). LDLT surgery was performed, and standard immunosuppressive therapy was administered postoperatively. Two weeks after liver transplantation, laboratory tests revealed significant improvement, with a noticeable reduction in jaundice, indicating that liver transplantation has a substantial therapeutic effect on PFIC3 patients. According to a report by Anna Maria Kavallar et al. [13], the 5-year post-transplant survival rate for PFIC3 patients was 97.6%, with a 5-year graft survival rate of 98.2%. Additionally, 2.9% of patients (3 out of 105) required re-transplantation, although the aetiology was not specified. Notably, in our study, Patient 3 died two years post-surgery while awaiting a bone marrow transplant due to PTLD. PTLD, a disease characterized by abnormal lymphocyte proliferation due to immunodeficiency following solid organ or hematopoietic cell transplantation, has a poor prognosis and can be life-threatening [14]. The incidence of PTLD is closely associated with EBV infection. Liver transplant patients with preexisting autoimmune hepatitis or immune disorders are at significantly increased risk of developing PTLD [15]. Early onset of EBV DNAemia less than 6 months post-transplant in donors under 5 years of age and who are CMV seronegative are independent risk factors for chronic high EBV load carriers [16]. Recent studies by Hannes Vietzen et al. [17] have demonstrated that the HLA-E-mediated immune response plays a critical role in controlling Epstein-Barr virus infection and preventing lymphoproliferative diseases post-transplantation. Exploring new and effective treatment methods is necessary for patients who are unresponsive to traditional therapies. Susan Prockop et al. investigated a novel immunotherapy [18] in which readily available EBV-specific T cells were used to treat EBV-associated lymphomas that did not respond to rituximab post-transplant. These findings indicate that this immunotherapy effectively identifies and eradicates EBV-infected cells, thereby controlling lymphoma progression. Clinical trials revealed that this therapy has high safety and efficacy, offering a new treatment option for post-transplant patients, particularly those unresponsive to conventional treatments.

With respect to liver histopathology, all the children displayed characteristics of biliary cirrhosis, including portal vein fibrosis, bile duct injury, inflammatory cell infiltration, and cholestasis, which aligns with the liver disease report. These findings further substantiate the pathophysiological process of PFIC3, namely, that ABCB4 gene mutation results in cholestasis and liver damage. MDR3, which is expressed on the tubule side of hepatocytes, exhibits altered expression patterns that are uncommon in other cholestatic liver diseases, making it a valuable diagnostic marker for PFIC3. MDR3 immunohistochemical staining revealed that MDR3 expression was reduced in the liver tissues of child 1, but was absent in the liver tissues of child 2 and child 3. This finding indicates that, although the MDR3 protein is present in the liver cells of child 1, its level is lower than normal, which might be attributed to partial loss of function due to ABCB4 gene mutation. The MDR3 protein was not detected in the liver cells of the other two children, possibly because a more severe ABCB4 gene mutation that led to complete failure of protein expression or total loss of function of the expressed protein. This functional deficiency culminates in cholestasis and biliary cirrhosis. This insight offers a clear path for exploring more therapeutic approaches in the molecular field. The regulation of ABCB4 expression on the tubule membrane is mediated by the C-terminal QNL motif, which serves as a top membrane retention motif through its interaction with EBP50 [19]. On the tubule plasma membrane, the activity of ABCB4 is modulated by the phosphorylation of several residues [20].

PFIC3 disease is attributed to a mutation in the ABCB4 gene, which leads to a loss or reduction in the expression of the MDR3 protein. This disruption hinders the efficient transport of phosphatidylcholine from liver cells to bile, ultimately resulting in disease. In the field of molecular genetics, we identified five mutations in the ABCB4 gene through genetic testing of three children, including three novel mutation sites. The two newly discovered heterozygous mutation sites in child 1 were C.2493G > C (p.R831S) and C.592G > A (p.A198T). According to ACMG guidelines, both of these mutations are classified as having uncertain clinical significance. The evidence is presented in Table 2. We hypothesize that protein function may be partially retained; however, over time, liver fibrosis and liver function impairment gradually worsen, eventually leading to the onset of PFIC3 disease. Child 2 carries a homozygous gene mutation c.3136C > T, which has been preliminarily determined to be pathogenic according to ACMG guidelines. The evidence is shown in Table 2. The patient was previously diagnosed with cholesterol gallstone disease [8], and considering the clinical manifestations of child 2 and the results of immunohistochemical analysis showing loss of MDR3 expression, we believe that the diagnosis of PFIC3 is justified. The two newly identified heterozygous mutation sites in child 3 were c.938C > T (p.A313V) and c.475C > T (p.R159X). According to ACMG guidelines, the mutation c.938C > T (p.A313V) was preliminarily classified as of uncertain clinical significance, whereas c.475C > T (p.R159X) was preliminarily determined to be pathogenic. The evidence is shown in Table 2, and the disease was diagnosed as PFIC3 in previous reports [9]. The age of onset for the patient in this case was 54 months, which is comparable to that of child 3 at 47 months. Furthermore, the absence of MDR3 expression via immunohistochemistry suggests that the same genetic phenotype is correlated with a similar clinical phenotype.

Although the pathophysiological process of PFIC3 was thoroughly investigated in this study, several limitations remain. First, the sample size was small and limited to patients at a single medical center, which may introduce selection bias. Second, our study did not include long-term follow-up of the children, necessitating further long-term studies to evaluate the efficacy of liver transplantation in patients with PFIC3. Additionally, although the expression of MDR3 protein has been preliminarily analysed, the impact of different mutation types on protein structure and function requires further investigation.

In summary, the present study provides new insights into the relationships between genetic mutations in PFIC3 and clinical features, genotypic phenotypes, and hepatic pathological features, and we report the adverse consequences of these mutations resulting from childhood onset, which we hope will help to improve the early recognition and understanding of this disease. Ursodeoxycholic acid is effective in patients with residual MDR3 but does not prevent progression of PFIC3 disease. Liver transplantation currently remains the only effective treatment strategy for end-stage liver disease. With the advancements in parental liver transplantation and the progress in post-transplant immunosuppressive therapy in recent years, an increasing number of children with end-stage liver disease have benefited. Therefore, for patients with a family history or suspected PFIC3, genetic testing should be conducted as early as possible for a definitive diagnosis. Simultaneously, for diagnosed patients, a personalized treatment plan should be developed to enhance treatment outcomes and quality of life.

## Conclusion

Early childhood PFIC3 significantly impacts the growth and health of children. Immunohistochemical and molecular genetic analysis of MDR3 is essential for the early and accurate diagnosis of PFIC3 in children. Early diagnosis and treatment can provide better therapeutic outcomes for affected children. In recent years, the development of parental liver transplantation technology has brought new hope to children with end-stage liver disease.

## Data Availability

All data supporting the findings of this study are available within the article and its Supplemental Information files. The data generated and analyzed during the present study are available from the corresponding author upon reasonable request.

## Abbreviations

ABCB4: ATP binding cassette subfamily B member 4
ALT: Alanine aminotransferase
AST: Aspartate aminotransferase
DBIL: Direct bilirubin
γ-GGT>: γ-Glutamyl transferase
IBIL: Indirect bilirubin
LDLT: Living donor liver transplantation
MDR3: Multidrug resistance protein 3
NH₃: Blood ammoni
PFIC3: Progressive familial intrahepatic cholestasis type 3
PC: Phosphatidylcholine
PLT: Platelet count
PTLD: Lymphoproliferative disorders post-transplantation
TBA: Total bile acid
TBIL: Total bilirubin
UDCA: Ursodeoxycholic acid
